# Differential Expression of Circulating Inflammatory Proteins Following Sport-Related Traumatic Brain Injury

**DOI:** 10.3390/ijms21041216

**Published:** 2020-02-12

**Authors:** Ghazala Begum, Rahul Reddy, Kamal M. Yakoub, Antonio Belli, David J. Davies, Valentina Di Pietro

**Affiliations:** 1 Marker Diagnostics Ltd., The BioHub, Edgbaston, Birmingham B15 2SQ, UK; 2 University of Illinois, Chicago, IL 60607, USA; rahulr3@illinois.edu; 3 Neuroscience & Ophthalmology Research Group, Institute of Inflammation & Ageing, College of Medical and Dental Sciences, University of Birmingham, Edgbaston, Birmingham B15 2TT, UK; K.Yakoub@bham.ac.uk (K.M.Y.); A.Belli@bham.ac.uk (A.B.); V.DiPietro@bham.ac.uk (V.D.P.); 4 National Institute for Health Research Surgical Reconstruction and Microbiology Research Centre, Queen Elizabeth Hospital, Edgbaston, Birmingham B15 2TH, UK

**Keywords:** mild traumatic brain injury, concussion, neuroinflammation, FGF21, MCP-1

## Abstract

Sport-related traumatic brain injury (TBI) elicits a multifaceted inflammatory response leading to brain injury and morbidity. This response could be a predictive tool for the progression of TBI and to stratify the injury of which mild TBI is most prevalent. Therefore, we examined the differential expression of serum inflammatory markers overtime and identified novel markers in repetitively concussed athletes. Neuropsychological assessment by Wechsler Adult Intelligence Scale (WAIS) and Immediate Post Concussion Assessment and Cognitive Test (ImPACT) was performed on rugby players and serum was taken from healthy, concussed and repetitively concussed athletes. Serum was also obtained <1 week and >1 week after trauma and analyzed for 92 inflammatory protein markers. Fibroblast growth factor 21 (FGF21) and interleukin-7 (IL-7) differentiated repetitively concussed athletes. Macrophage chemotactic protein-1 (MCP-1), tumor necrosis factor superfamily member 14 (TNFSF14) were significantly reduced >1 week and chemokine (C-X3-C motif) ligand 1 (CX3CL1) upregulated <1 week after injury. FGF21 and MCP-1 negatively correlated with symptoms and their severity. We have identified dynamic changes in the inflammatory response overtime and in different classes of concussion correlating with disease progression. This data supports the use of inflammatory biomarkers as predictors of symptom development due to secondary complications of sport-related mTBI.

## 1. Introduction

Traumatic brain injury (TBI) causes damaging neurological impairments leading to disability and morbidity. TBI affects young and old, with young victims creating a substantial negative impact to families and wider society with years of lost productivity and an increasing burden on healthcare systems [1,2]. Such is its prevalence that its economic cost to the US is $56 billion a year [3]. This large socio-economic burden can lead to poor post trauma follow up exacerbating a patient’s injuries. Therefore, there is a need to not only identify therapies, but to also have accurate diagnostic and prognostic monitoring capabilities. 

Mild TBI (mTBI) is the most prevalent form of TBI with up to 80% of patients suffering with adverse symptoms such as sleep disturbances, headaches, nausea, impaired memory, or loss of function [4]. mTBI can be defined as head trauma resulting in either memory loss or altered neurological function up to a day following the traumatic ictus or loss of consciousness for under 30 min [5]. A large proportion of mTBI consists of sport-related concussive (SRC) injury with up to 3.8 million cases reported in the United States annually [6]. There is also a greater risk of these athletes experiencing repetitive concussions which has been associated with the development of neurodegenerative diseases such as Parkinson’s, Alzheimer’s, and chronic traumatic encephalopathy (CTE) [6,7,8,9,10,11,12,13,14,15]. Neurocognitive tests have been developed to diagnose concussion. However, these assessments are expensive, subject to bias due to lack of appropriate baselines and have not yet demonstrated efficacy as prognostic tools [16,17,18]. 

Difficulties in developing tools for the prediction of progressive pathology in TBI are confounded by the variability in the anatomical location, extent of the mechanical injury to the brain, as well as the multitude of biological response mechanisms involved. TBI is broadly characterized by 2 phases [19,20]. Immediately after impact, the primary phase is associated with shearing and tearing of neural and vascular brain structures and enhanced permeability of the blood–brain barrier. Within minutes start the secondary phase, where danger-associated molecular pattern (DAMPs) molecules are released from damaged cells which stimulate the Toll-like receptor (TLR) [21]. Cytokines and chemokines are also released leading to the recruitment of microglia and peripheral monocytes to the site of injury [22]. 

In addition, cytotoxic and ischemic responses enhance axonal death and ultimately injury to neurological structures [21]. This is associated with the conversion of monocytes to macrophages and the T cell production of cytokines. Macrophages are important in removing debris however, they can have a dual effect of allowing tissue repair or additional damage [22]. The inflammatory changes can remain for days to months exacerbating injury in systemic and intracranial systems. 

Simultaneously, microglia potentiate neuronal recovery through the production of pro- and anti-inflammatory mediators [23]. This includes the interleukins IL-6 and IL-7 as well as tumor necrosis factor-α (TNF-α) and chemokines macrophage chemotactic protein-1 (MCP-1). Consequently, they display both a neuroprotective and neurodegenerative response [24]. This interplay continues to change and evolve as the secondary response progresses, ultimately leading to increased expression of reactive oxygen species (ROS) in microglia and further cell injury [25]. Therefore, targeting the inflammatory system could provide a potential therapeutic strategy for minimizing post-injury evolution and improving survival [26].

In addition to potential therapies, inflammatory proteins could also be used for the development of biomarkers useful in the clinical assessment of mTBI. Efforts have been focused on quantifying inflammatory markers in easily obtained bodily fluids such as serum, saliva, and urine. It has already been shown that IL-6 was differentially expressed in serum levels of TBI patients within hours after injury [27] and IL-10 is strongly associated with morbidity [28]. Following extensive protein analysis Cystatin D, AXIN1, and TRAIL were also identified as biomarkers for the early detection of TBI and were further able to differentiate between mild and severe TBI [29]. In addition to immediate changes, inflammatory markers could also be used to track recovery over time in severe traumatic injuries [30]. 

However, studies investigating the relationship between inflammatory markers and the ability to track the evolution of both symptoms and physiological responses to sports-related concussion (SRC) over time, are limited. Moreover, little has been done to assess the differential expression of the circulating inflammatory response in athletes who have suffered repetitive concussions. 

Consequently, in this study we have focused analysis on inflammatory markers in the serum of semi-professional and professional Rugby players who suffered mTBI and repeated mTBI. These samples were obtained less than one week after injury (<1 week) to determine the early inflammatory response and over 1 week (>1 week) following injury to assess longitudinal inflammatory effects. In addition to investigating the inflammatory network in athletes after a single concussion (C), those who suffered repeated concussions (RC) were also investigated. This data has been analyzed in conjunction with and contextualized in relation to cognitive performance and symptom severity, with protein expression as a predictive tool for the evolving inflammatory response seen in mTBI.

## 2. Results

### 2.1. Identifiers of Concussed and Repetitively Concussed Athletes 

**Figure 1:**
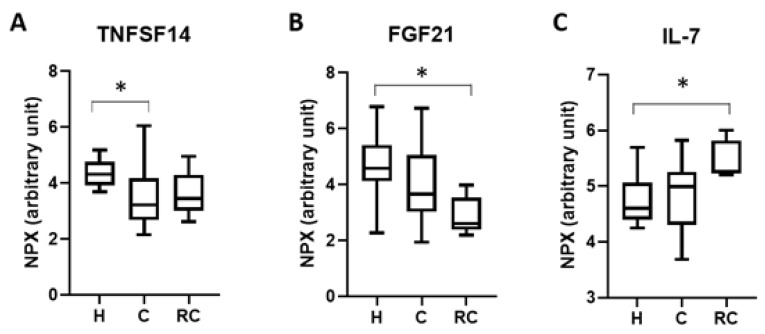
Differentially expressed proteins in human serum from healthy (H), concussed (C), and repetitively concussed (RC) patients. TNFSF14 (**A**) was found to be significantly reduced in concussed patients when compared to healthy individuals. In contrast FGF21 (**B**) was significantly downregulated and IL-7 (**C**) was significantly upregulated in repetitively concussed patients. Data is presented as the mean ± SE and was tested for normality followed by a one-way Anova and a tukey post hoc test where * *p* < 0.05. H = 12 C = 18 RC = 5.

The levels of 92 inflammatory markers were measured in the serum from H, C, and RC athletes whose samples were collected within 2–14 days following their most recent concussion. RC athletes were those players that had received 2 concussions within a 3-month period. Following this analysis, the 3 proteins tumor necrosis factor superfamily member 14 (TNFSF14), fibroblast growth factor 21 (FGF21), and interleukin 7 (IL-7) were found to be altered between the groups (Figure 1). TNFSF14 levels were significantly reduced in C patients when compared to H and RC groups (Figure 1A; H = 4.38 ± 0.52, C = 3.44 ± 1.10, RC = 3.61 ± 0.85; H vs. C *p* = 0.0278). Following the same analysis two clear markers were identified for RC athletes differentiating them from healthy and single concussion groups. In these occurrences FGF21 was significantly downregulated in RC serum (Figure 1B; H = 4.65 ± 1.25, C = 4.05 ± 1.32, RC = 2.89 ± 0.69; H vs. RC *p* = 0.037) and IL-7 was significantly increased in RC athletes (Figure 1C; H = 4.78 ± 0.45, C = 4.83 ± 0.59, RC = 5.47 ± 0.35; H vs. RC *p* = 0.043, C vs. RC *p* = 0.049).

### 2.2. Differential Expression of Biomarkers at Early and Late Timepoints Following Concussion

**Figure 2:**
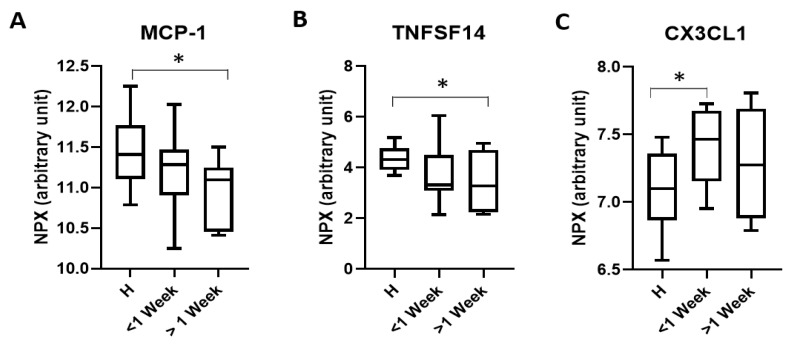
Changes in serum protein levels following analysis under (<1 week) and over a week (>1 week) after injury when compared to healthy (H) athletes. MCP-1 (**A**) and TNFSF14 (**B**) levels were significantly reduced in serum taken from patients >1 week after injury. CX3CL1 (**C**) was significantly upregulated in serum taken <1 week following injury when compared to H. Data is presented as the mean ± SE and was tested for normality followed by a one-way Anova and a tukey post hoc test where * *p* < 0.05. H = 12, <1 week = 11, >1 week = 7.

In order to determine if there are any changes in biomarkers indicative of ongoing mTBI pathology in the extended post-injury time window, we compared the expression of markers in athletes less than a week following concussion and those over a week (average 60 days following injury). Three markers were identified at the different time points. Both MCP-1 (Figure 2A; H = 11.46 ± 0.43, <1 week = 11.16 ± 0.50, >1 week = 10.90 ± 0.41; H vs. >1 week *p* = 0.030) and TNFSF14 (Figure 2B; H = 4.38 ± 0.52, <1 week = 3.56 ± 1.02, >1 week = 3.28 ± 1.03; H vs. >1 week *p* = 0.038) were significantly reduced in the serum of patients >1 week after the initial injury when compared to controls. The only marker found to be significantly altered in patients that presented <1 week after injury was the chemokine (C-X3-C motif) ligand 1 (CX3CL1) which was increased when compared to H participants (Figure 2C; H = 7.07 ± 0.29, <1 week = 7.43 ± 0.39, >1 week = 7.32 ± 0.37, *p* = 0.027).

### 2.3. Clinical Assessment of Secondary Symptoms Following mTBI

**Figure 3:**
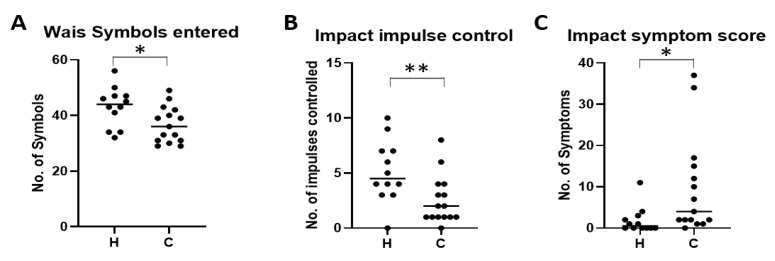
Outcomes of the Wechsler Adult Intelligence Scale (WAIS) symbol search and Impact test for concussion for the patients within this study. Following comprehensive clinical assessment of the WAIS symbol search and Impact test on healthy (H) and concussed (C) athletes it was found that the number of symbols entered (**A**), the impact on impulse control (**B**) and the impact symptom score (**C**) were significantly altered. Data is represented as the mean and individual data points were data was analyzed for significance using the unpaired T test where * *p* < 0.05, ** *p* < 0.01. WAIS symbol search *n*: H = 12, C = 15. Impact test *n*: H = 12 C = 15.

Concussed patients displayed a range of symptoms with headaches being the most commonly reported at 63% (Table 1: *n* = 51 patients). This was followed by fatigue (29%), fogginess (29%), drowsiness (27%), and having trouble falling asleep (27%). Patients were clinically assessed by the WAIS symbol search and ImPACT tests. Despite the range of criteria that the WAIS assessment covered only the number of symbols entered was found to be significantly lower in concussed patients (Figure 3A; H = 43.17 ± 7.07, C = 3 6.67 ± 6.45, *p* = 0.0196). The ImPACT test revealed that the amount of impulse control was significantly reduced in concussed patients (Figure 3B; H = 5.167 ± 2.79, C = 2.533 ± 2.20, *p* = 0.0110) and their symptom score was higher when compared to controls (Figure 3C; H = 1.833 ± 3.19, C = 9.733 ± 11.79, *p* = 0.0336).

### 2.4. FGF21 and MCP-1 Correlate with Symptom Severity and Cognitive Performance

**Figure 4:**
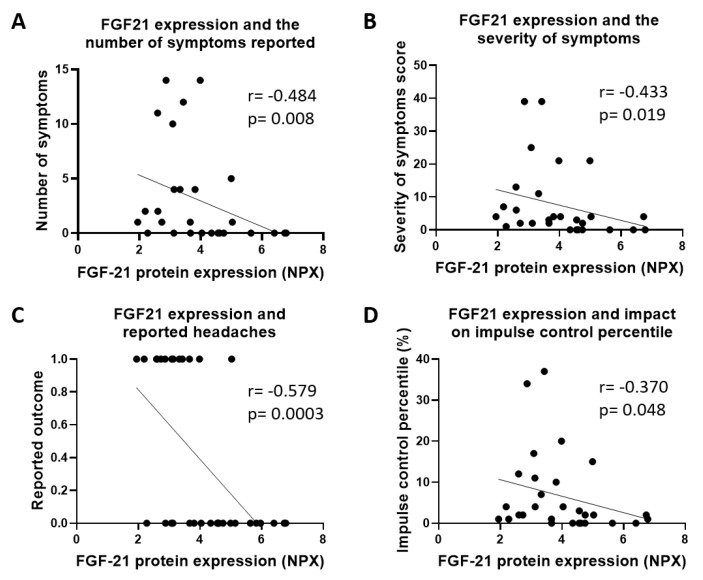
Fibroblast growth factor 21 (FGF21) protein expression correlated to clinical data. Reduced FGF21 protein expression was associated with increases in the (**A**) number and (**B**) severity of the symptoms such as (**C**) headaches. There was also a correlation of low levels of FGF21 and the (**D**) impact on impulse control. Data was analyzed according to Spearman’s correlation coefficient where the number of pairs = 29 and *p* < 0.05.

**Figure 5:**
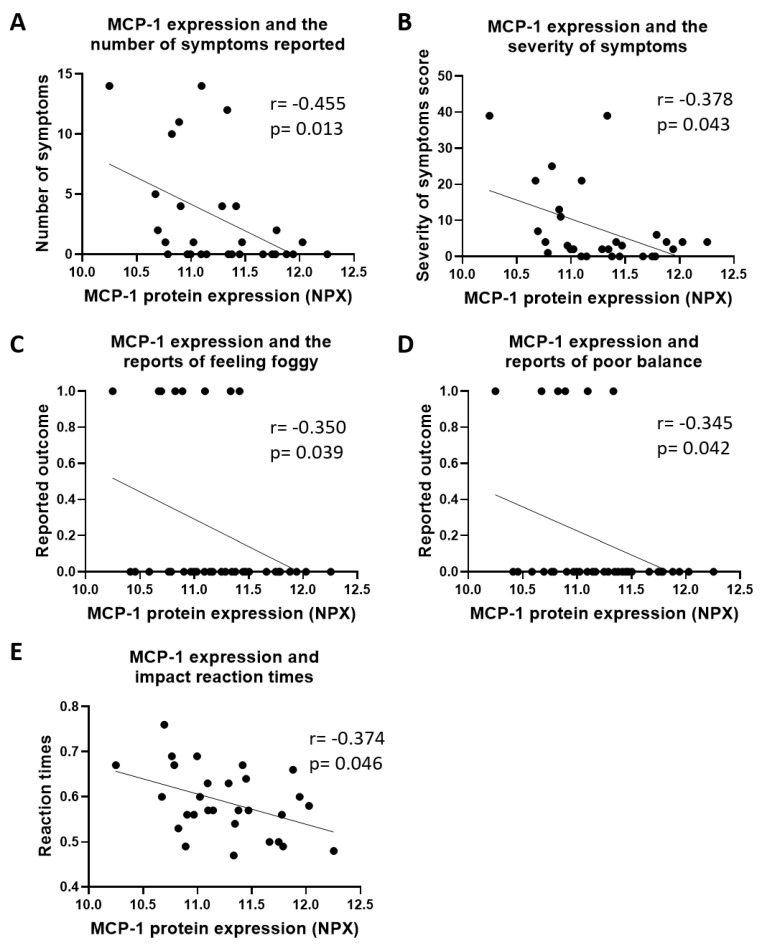
Macrophage chemotactic protein-1 (MCP-1) protein expression correlated to clinical data. Reduced MCP-1 expression was associated with increases in the number of (**A**) reported symptoms and their (**B**) severity. This included feeling (**C**) foggy and reports of (**D**) poor balance. There was also a significant negative correlation between MCP-1 expression and (**E**) reaction times. Data was analyzed according to Spearman’s correlation coefficient where the number of pairs = 29 and *p* < 0.05.

To determine if the expression of inflammatory proteins correlates with clinical symptoms of secondary TBI all proteins were compared to clinical neuropsychological data using Spearman’s correlation coefficient. Of these proteins, serum expression of FGF21 (Figure 4) and MCP-1 (Figure 5) were the most indicative of clinical characteristics. Low levels of circulating FGF21 was associated with an increase in the number of reported symptoms (Figure 4A; *r* = −0.484, *p* = 0.008) as well as greater severity of the symptoms (Figure 4B; *r* = −0.433, *p* = 0.019). Of these symptoms reported headaches showed a significant negative correlation with protein expression (Figure 4C; *r* = −0.579, *p* = 0.0003). FGF21 expression was also negatively correlated with a larger impact on impulse control (Figure 4D; *r* = −0.370, *p* = 0.048).

Reduced serum levels of MCP-1 were also related to an increase in the number (Figure 5A; *r* = 0.455, *p* = 0.013) and severity of symptoms (Figure 5B; *r* = −0.378, *p* = 0.043) reported. Low levels of MCP-1 were correlated with greater numbers of patients feeling foggy (Figure 5C; *r* = −0.350, *p* = 0.039) and having poor balance (Figure 5D; *r* = −0.345, *p* = 0.042). Additionally, reduced serum MCP-1 levels were found to be consistent with increases in the time to react (Figure 5E; *r* = −0.374, *p* = 0.046).

## 3. Discussion

In this study we have provided further evidence of circulating inflammatory biomarkers correlating with symptoms following mTBI in the serum of athletes who have had multiple concussions. Many investigations have analyzed the immediate inflammatory response after TBI [19,20,21]. This data may aid our understanding of the complex mechanism following mTBI leading to the development of accurate diagnostic and prognostic tools to better define the period of injury and guide a safe return to play objective protocol. 

The injury burden of mTBI may have potentially caused the participants in our study to exhibit reduced cognitive performance such as impaired memory and impulse control as well as the development of symptoms including headaches, fatigue and sleep disturbances. Processing speed and visual processing was measured by the WAIS IV symbol search which is an assessment of head injury of all etiologies, we also utilized the computerized ImPACT test which was developed for athletes [18,31] in this investigation. This allowed us to identify changes in motor speed, reaction time and impulse control which are key components in the ImPACT assessment. However, whilst we observed some changes in cognitive performance the validity of the test for clinical use is still unclear and therefore, as others have found it may not be accurate enough for diagnosis of mTBI alone [18,32,33,34].

The secondary inflammatory response following concussion is regulated by pro- and anti-inflammatory cytokines [21,22]. Expression patterns of these cytokines is associated with the different forms and severity of TBI [29,35]. In our study we were able to assess variances in the expression of 92 circulating inflammatory proteins in athletes suffering from repetitive concussions within a three-month period. Of these candidate markers FGF21 and IL-7 were significantly altered. FGF21 is important in the process of cell proliferation including that of oligodendrocyte precursor cells (OPC) [36] and is involved in oligodendrocyte development and remyelination [37,38]. In a murine model of TBI, FGF21 was able to protect the blood–brain barrier by forming a complex with its receptors [39]. It has also been shown to enhance angiogenesis and the restoration of functional anatomy in the brain [40]. Consequently, a decrease in circulating FGF21 following repetitive concussion could lead to deleterious effects in cerebral tissue. This was evidenced by the significant negative correlation of FGF21 protein expression and the number of symptoms and their severity including headaches as reported in our study. A central neurological affect is further supported by studies within the literature that have observed FGF21 crossing the blood–brain barrier [41]. In addition to its potential as a biomarker for repetitive concussion and its potential cumulative effect on neurological pathology, FGF21 could be an effective therapeutic strategy for the guidance treatment of repetitive TBI.

We demonstrate that IL-7 was also significantly increased in the RC athletes when compared to controls. This protein is important for the development and survival of T-lymphocytes and it can enhance the production of pro-inflammatory cytokines [42]. Interestingly increased IL-7 in the serum has been found in patients with multiple sclerosis [43], a demyelinating disease of the central nervous system. This could be due to IL-7s ability to upsurge the accumulation of activated microglia/macrophages leading to secondary damage and regeneration failure [44]. Therefore, it could be hypothesized that augmented levels of IL-7 in RC patients could be a poor prognostic indicator in their recovery, as has been evidenced in another study where increased IL-7 was a positive prognostic factor in the development of posttraumatic depression following TBI [45]. 

The protein TNFSF14, was the only marker able to differentiate between concussed and repetitively concussed patients. In C patient cohort, the decrease in TNFSF14 serum expression was also found to be attenuated overtime. This protein is known to trigger apoptosis in tumor cells as well as regulating the proliferation of T cells [46,47,48]. TNFSF14 has been identified as a risk gene for multiple sclerosis [49] however, little is known about its affects in TBI and further investigation of this relationship is required. 

We also investigated changes in protein expression overtime in athletes that had received a single concussion. Our data showed significant changes in MCP-1 (also known as CCL2), which is one of the most well correlated TBI proteins [35,50]. MCP-1 has been shown to be expressed in the CNS by astrocytes [51,52]. It can attract cells including macrophages and microglia via inflammatory stimuli [50]. This recruitment enhances the inflammatory response leading to further degeneration and as such central inhibition of MCP-1 has been shown to reduce neuronal loss [53]. Many investigations have shown an increase in MCP-1 expression within 5 days following TBI [50,52,54]. In contrast to this, we found serum MCP-1 levels attenuated overtime negatively correlating with patients feeling foggy and having poor balance. The decrease in MCP-1 could be a protective reaction preventing further neurological injury. However, most of these studies have been carried out in animal models over shorter periods of time following injury. They were also carried out in a range of different levels and methods of injury. 

Differential expression of the chemokine CX3CL1 (also known as Fractalkine) was also found. CX3CL1 has been shown to have an important role in the CNS where it is present in many areas including the cerebral cortex, amygdala, and the hippocampus [55]. In these areas it maintains the dynamics between neurons and microglia [56]. During neurotoxicity, CX3CL1 has been found to protect microglia by stimulating anti-apoptotic Bcl in addition to producing a phagocytic response to inhibit neurotoxic stimuli [57]. Previous studies have shown changes in CX3CL1 in both the CSF and the serum of patients suffering TBI however, there are conflicting outcomes in whether its expression increases of decreases [58,59]. In our study the increase observed within a week after injury could be indicative of CX3CL1 acting to increase microglia activity in response to neurotoxicity. CX3CL1 therefore, is a potential biomarker and target for therapeutic intervention in mTBI. However, the data presented in this manuscript is within the discovery phase and all the candidate markers would need to be further investigated within a larger cohort to determine their suitability as biomarkers of mTBI.

We have identified changes in the expression of inflammatory proteins that could be further developed as diagnostic tools for the resolution of the evolving injury within sports-related mTBI patients and in those who have suffered repetitive concussion. This included FGF21 and MCP-1 whose expression negatively correlated with symptom severity and cognitive performance. These proteins all have an emerging role in the pathogenesis of mTBI and could also be utilized for therapeutic advancement.

## 4. Materials and Methods 

### 4.1. Study Approval

Study participants were recruited through the Birmingham concussion clinic at the Queen Elizabeth Hospital Birmingham (QEHB) or at the University of Birmingham (UoB). (UK), as part of the RECOS (The REpetitive COncussion in Sport) (Ethics Ref. REC 17/EE/0275, 22 September 2017) [60]. Written informed consent was received from participants prior to inclusion in the study.

### 4.2. Recruitment

Male and female athletes aged 16–34 years, participating in professional and semi-professional Rugby who have been positively diagnosed as having a concussion along with a normal neurological objective examination at assessment, were enrolled in this study. In addition to those that had suffered single concussions, we also recruited athletes that had suffered 2 concussions within a 3-month period. These athletes had an initial concussive event followed by another one within 3 months. Following their last concussion, samples were taken when they first came to the clinic, between 2–14 days. Individuals who require hospital admission after initial assessment for their TBI, presenting intracranial blood, brain tissue injury, or non-TBI related pathologies on initial CT/MR scan, any history of neurodegenerative pathology or history of chronic alcohol or drug abuse were excluded. In addition, age matched controls, who have not received any concussion in the previous 3 months, were recruited as healthy controls (H). Blood samples were taken <1 week after or >1 week after injury. Of these samples, one athlete had taken paracetamol 24 h before entering the clinic. 

Clinical, demographic (Table 2), neuropsychometric and imaging parameters were collected for each subject on the day that they came to report their concussions to the clinic, including, among others, parameters forming part of The Sport Concussion Assessment Tool—5th Edition (SCAT 5) (e.g., symptoms inventory, injury data, balance errors, and immediate memory and recall tests) [61], concussion history, the Immediate Post-Concussion Assessment and Cognitive Testing battery (ImPACT-FDA approved computerized neuropsychometric suite for sport concussion) [31], and the Wechsler Adult Intelligence Scale version IV symbol search module (WAIS-IV symbol search) [32].

### 4.3. Blood Collection and Processing

Samples were collected from non-concussed healthy athletes, athletes who had suffered a concussion with Peripheral venous blood samples were obtained from patients following admission between 2–5 days (<1 week) and 15–75 days (>1 week) after the initial trauma and processed within 2 h after venepuncture. Samples were kept at room temperature for 30 min after which they were centrifuged at 3000 rpm for 10 mins at 4 °C. Serum was separated and stored at −80 °C until further use. 

### 4.4. Multiplex Protein Assay

Serum expression of 92 inflammatory markers were analyzed using the Proseek Multiplex Inflammation I assay (Olink Bioscience, Uppsala, Sweden) as described previously (Table 3; 29). Briefly, according to the manufacturers protocol, 1 µL of serum was incubated at 8 °C overnight with antibodies labelled with DNA nucleotides. This was then combined with and extension mix in a PCR plate. These plates underwent a 5′ incubation followed by 17 cycles of DNA amplification. Samples were added to a detection mix and loaded onto a primed 96.96 Dynamic Array IFC (Fluidigm, CA, USA) and read in the Fluidigm Biomark reader. Data were then analyzed using the Olink Wizard for GenEx software (Olink) and calculated from Ct values. Protein expression were normalized and presented as normalized protein expression (NPX). NPX units are inverted to Ct values and as such high NPX values are indicative of high protein levels. 

### 4.5. Statistical Analysis

All data is presented as the mean ± standard deviation. Comparison of protein expression between the control and concussion groups and the time points was first tested for distribution using the Shapiro Wilks test after which a one-way ANOVA with Tukey post hoc test was performed, where *p* < 0.05. To identify correlations between protein expression and cognitive performance and symptoms detailed in the WAIS and ImPACT tests, data were analyzed using the Spearman’s correlation coefficient where *p* < 0.05. In order to generate correlations with symptoms the presentation of a symptom was scored as 1 and where the symptom was not apparent it was scored as 0. All data was analyzed using GraphPad Prism 8 (GraphPad Inc., San Diego, CA, USA).

## Figures and Tables

**Table 1:** The table represents the most common symptoms reported by patients following concussion in the study. Headaches were highly reported followed by fatigue, feeling foggy, drowsiness, and having trouble falling asleep. In a few cases nausea was also present. Data is presented as a percentage of total number of patients. Concussed = 51.

Symptoms	Concussed (Number of Patients Reporting the Symptom %)
Headache	63
Fatigue	29
Foggy	29
Drowsiness	27
Trouble falling asleep	27
Balance	23
Dizziness	19
Irritability	19
Nausea	10

**Table 2:** Patient demographics. H = healthy controls, C = Single concussion, RC = repetitively concussed, <1 week = athletes with a single concussion analyzed less than one week from injury, >1 week = athletes with a single concussion analyzed more than one week from injury.

Characteristics	H (*n* = 12)	C (*n* = 18)	RC (*n* = 5)	<1 Week (*n* = 11)	>1 Week (*n* = 7)
Age (years; Mean ± SD)	25.5 ± 5.9	27.2 ± 4.9	24.5 ± 3.2	28.4 ± 4.9	24.4 ± 4.1
Gender (M/F)	10/2	17/1	4/1	11/0	6/1

**Table 3:** 92 inflammatory markers contained within the Proseek Multiplex inflammation assay (Olink Bioscience, Uppsala, Sweden).

Adenosine Deaminase (ADA)	Caspase 8 (CASP-8)	Interleukin-12 subunit beta (IL-12B)	Latency-associated peptide transforming growth factor beta 1 (LAP TGF-beta-1)	STAM-binding protein (STAMPB)
Artemin (ARTN)	CUB domain-containing protein 1 (CDCP1)	Interleukin-13 (IL-13)	Leukemia inhibitory factor (LIF)	Stem cell factor (SCF)
Axin-1 (AXIN1)	Cystatin D (CST5)	Interleukin-15 receptor subunit alpha (IL-15RA)	Leukemia inhibitory factor receptor (LIF-R)	Sulfotransferase 1A1 (ST1A1)
Beta-nerve growth factor (Beta-NGF)	Delta and Notch-like epidermal growth factor-related receptor (DNER)	Interleukin-17A (IL-17A)	Macrophage colony-stimulating factor 1 (CSF-1)	T cell surface glycoprotein CD6 isoform (CD6)
Brain-derived neurotrophic factor (BDNF)	Eotaxin-1 (CCL11)	Interleukin-17C (IL-17C)	Matrix metalloproteinase-1 (MMP-1)	T-cell surface glycoprotein CD5 (CD5)
C-C motif chemokine 19 (CCL19)	Eukaryotic translation initiation factor 4E-binding protein 1 (4E-BP1)	Interleukin-18 (IL-18)	Matrix metalloproteinase-10 (MMP-10)	Thymic stromal lymphopoietin (TSLP)
C-C motif chemokine 20 (CCL20)	Fibroblast growth factor 19 (FGF-19)	Interleukin-18 receptor 1 (IL-18R1)	Monocyte chemotactic protein 1 (MCP-1)	TNF-beta (TNFB)
C-C motif chemokine 23 (CCL23)	Fibroblast growth factor 21 (FGF-21)	Interleukin-2 (IL-2)	Monocyte chemotactic protein 2 (MCP-2)	TNF-related activation-induced cytokine (TRANCE)
C-C motif chemokine 25 (CCL25)	Fibroblast growth factor 23 (FGF-23)	Interleukin-2 receptor subunit beta (IL-2RB)	Monocyte chemotactic protein 3 (MCP-3)	TNF-related apoptosis-inducing ligand (TRAIL)
C-C motif chemokine 28 (CCL28)	Fibroblast growth factor 5 (FGF-5)	Interleukin-20 (IL-20)	Monocyte chemotactic protein 4 (MCP-4)	Transforming growth factor alpha (TGF-alpha)
C-C motif chemokine 3 (CCL3)	Fms-related tyrosine kinase 3 ligand (Flt3L)	Interleukin-20 receptor subunit alpha (IL-20RA)	Natural killer cell receptor 2B4 (CD244)	Tumor necrosis factor (Ligand) superfamily, member 12 (TWEAK)
C-C motif chemokine 4 (CCL4)	Fractalkine (CX3CL1)	Interleukin-22 receptor subunit alpha-1 (IL-22 RA1)	Neurotrophin-3 (NT-3)	Tumor necrosis factor (TNF)
C-X-C motif chemokine 1 (CXCL1)	Glial cell line-derived neurotrophic factor (hGDNF)	Interleukin-24 (IL-24)	Neurturin (NRTN)	Tumor necrosis factor ligand superfamily member 14 (TNFSF14)
C-X-C motif chemokine 10 (CXCL10)	Hepatocyte growth factor (HGF)	Interleukin-33 (IL-33)	Oncostatin-M (OSM)	Tumor necrosis factor receptor superfamily member 9 (TNFRSF9)
C-X-C motif chemokine 11 (CXCL11)	Interferon gamma (IFN-gamma)	Interleukin-4 (IL-4)	Osteoprotegerin (OPG)	Urokinase-type plasminogen activator (uPA)
C-X-C motif chemokine 5 (CXCL5)	Interleukin-1α (IL-1α)	Interleukin-5 (IL-5)	Programmed cell death 1 ligand 1 (PD-L1)	Vascular endothelial growth factor A (VEGF-A)
C-X-C motif chemokine 6 (CXCL6)	Interleukin-10 (IL-10)	Interleukin-6 (IL-6)	Protein S100-A12 (EN-RAGE)	
C-X-C motif chemokine 9 (CXCL9)	Interleukin-10 receptor subunit alpha (IL-10RA)	Interleukin-7 (IL-7)	Signaling lymphocytic activation molecule (SLAMF1)	
CDL40 receptor (CD40)	Interleukin-10 receptor subunit beta (IL-10RB)	Interleukin-8 (IL-8)	SIR2-like protein 2 (SIRT2)	
